# Supplementary material for: Smoking Affects the Post-Stroke Inflammatory Response of Lipid Mediators in a Gender-Related Manner
Source: Biomedicines. 2022 Dec 29;11(1):92. doi: 10.3390/biomedicines11010092 (PMC9855814; doi:10.3390/biomedicines11010092)
Supplement: Supplementary file 1 [file biomedicines-11-00092-s001.zip › biomedicines-2007276-supplementary.pdf]

Supplementary Materials

Table S1. Characteristics of the study group.

Parameter	TOTAL NON-SMOKERS (TNS) Avg ± SD n = 46	TOTAL SMOKERS (TS) Avg ± SD n =27	p-value
Age (years)	61.4 ± 12.3	60.3 ± 13.1	ns
Height (cm)	168.2 ± 8.7	167.4 ± 8.1	ns
Weight (kg)	83.5 ± 16.6	76.6 ± 12.2	<0.05
BMI (kg/m^2)	29.4 ± 5.4	27.6 ± 4.2	ns

	WOMEN			MEN		
	NON-SMOKERS Avg ± SD n = 24	SMOKERS Avg ± SD n = 16	p-value	NON-SMOKERS Avg ± SD n = 22	SMOKERS Avg ± SD n = 11	p-value
Age (years)	62.0 ± 12.1	61.3 ± 13.7	ns	60.9 ± 77.0	58.8 ± 12.7	ns
Height (cm)	163.7 ± 6.9	162.1 ± 4.8	ns	173.2 ± 5.0	175.2 ± 5.2	ns
Weight (kg)	76.0± 14.7	69.9 ± 7.8	ns	91.7 ± 99.0	86.3 ± 11.0	ns
BMI (kg/m^2)	28.2 ± 4.8	27.3 ± 4.1	ns	30.7 ± 46.2	28.1 ± 4.5	ns

	SMOKERS			NON-SMOKERS		
	WOMEN Avg ± SD n = 16	MEN Avg ± SD n = 11	p-value	WOMEN Avg ± SD n = 24	MEN Avg ± SD n = 22	p-value
Age (years)	61.3 ± 13.7	58.8 ± 12.7	ns	62.0 ± 12.1	60.9 ± 77.0	ns
Weight (cm)	162.1 ± 4.7	175.2 ± 5.2	<0.01	163.7 ± 6.9	173.2 ± 5.0	<0.05
Height (kg)	69.9 ± 7.8	86.3 ± 11.0	<0.01	76.0 ± 14.7	91.7 ± 99.0	<0.05
BMI (kg/m^2)	27.3 ± 4.1	28.1 ± 4.5	ns	28.2 ± 4.8	30.7 ± 46.2	ns

Avg –mean, SD - standard deviation, ns - not statistically significant

Table S2. Schematic representation of involvement of individual metabolites AA, EPA, and DHA in post-stroke inflammatory response in women and men who smoked and did not smoke.

	WOMEN		MEN		SMOKER		NON-SMOKER	
	NON-SMOKER	SMOKER	NON-SMOKER	SMOKER	WOMEN	MEN	WOMEN	MEN
**LA metabolites**								
9S HODE	+	+	+	+	+	+	+	+
13S HODE	+	+	+	+	+	+	+	+
**AA metabolites**								
TXB2	+	+	+	+	+	+	+	+
Prostaglandin E2	-	+	+	-	+	-	-	+
LTX A4 5S, 6R	-	-	-	-	-	-	-	-
LTX A4 5S, 6R, 15R	-	-	-	+	-	+	-	-
Leukotriene B4	-	-	+	+	-	+	-	+
Leukotriene B4	-	-	+	+	-	+	-	+
15S HETE	+	+	+	+	+	+	+	+
12S HETE	+		+	+	-	+	+	+
5 HETE	+	+	+	+	+	+	+	+
5 oxo ETE	-	+	+	+	+	+	-	+
16RS HETE	-	-	-	-	-	-	-	-
**EPA metabolites**								
18RS HEPE	+	+	+	+	+	+	+	+
Resolvin E1	+	+	-	+	+	+	+	-
**DHA metabolites**								
Protectin D1	-	-	+	-	-	-	-	+
NPD1	+		+	+	-	+	+	+
Resolvin D1	-	-	+	-	-	-	-	+
Maresin 1	+	+	+	+	+	+	+	+
